# Quality control in scRNA-Seq can discriminate pacemaker cells: the mtRNA bias

**DOI:** 10.1007/s00018-021-03916-5

**Published:** 2021-08-24

**Authors:** Anne-Marie Galow, Sophie Kussauer, Markus Wolfien, Ronald M. Brunner, Tom Goldammer, Robert David, Andreas Hoeflich

**Affiliations:** 1 Institute of Genome Biology, Leibniz Institute for Farm Animal Biology (FBN), 18196 Dummerstorf, Germany; 2 Department of Cardiac Surgery, Rostock University Medical Center, 18057 Rostock, Germany; 3 Department of Life, Light, and Matter, Interdisciplinary Faculty, Rostock University, 18059 Rostock, Germany; 4 Department of Systems Biology and Bioinformatics, University of Rostock, 18051 Rostock, Germany; 5 Molecular Biology and Fish Genetics, Faculty of Agriculture and Environmental Sciences, University of Rostock, 18059 Rostock, Germany

**Keywords:** Conduction system, Sinoatrial node, Cardiomyocytes, Mitochondrial transcripts, Single-cell RNA-sequencing, Cluster analysis, iSABs

## Abstract

Single-cell RNA-sequencing (scRNA-seq) provides high-resolution insights into complex tissues. Cardiac tissue, however, poses a major challenge due to the delicate isolation process and the large size of mature cardiomyocytes. Regardless of the experimental technique, captured cells are often impaired and some capture sites may contain multiple or no cells at all. All this refers to “low quality” potentially leading to data misinterpretation. Common standard quality control parameters involve the number of detected genes, transcripts per cell, and the fraction of transcripts from mitochondrial genes. While cutoffs for transcripts and genes per cell are usually user-defined for each experiment or individually calculated, a fixed threshold of 5% mitochondrial transcripts is standard and often set as default in scRNA-seq software. However, this parameter is highly dependent on the tissue type. In the heart, mitochondrial transcripts comprise almost 30% of total mRNA due to high energy demands. Here, we demonstrate that a 5%-threshold not only causes an unacceptable exclusion of cardiomyocytes but also introduces a bias that particularly discriminates pacemaker cells. This effect is apparent for our in vitro generated induced-sinoatrial-bodies (iSABs; highly enriched physiologically functional pacemaker cells), and also evident in a public data set of cells isolated from embryonal murine sinoatrial node tissue (Goodyer William *et al.* in Circ Res 125:379–397, 2019). Taken together, we recommend omitting this filtering parameter for scRNA-seq in cardiovascular applications whenever possible.

**Supplementary Information:**

The online version contains supplementary material available at 10.1007/s00018-021-03916-5.

## Introduction

The advent of single-cell RNA-sequencing (scRNA-Seq) has permitted a deeper understanding of the complexity and heterogeneity of tissues. Employing microfluidic high-throughput scRNA-Seq platforms allowed for the discovery of previously unrecognized subpopulations with distinct gene expression profiles [2, 3]. Characterizing cardiac tissue, however, is problematic due to the delicate isolation process and the large size of mature cardiomyocytes that exceeds the diameter of the applied microfluidic systems. Facing these technical limitations, single-nucleus sequencing (snRNA-Seq) was established as commonly accepted alternative to study postnatal hearts despite omitting cytoplasmic and mitochondrial mRNA species [4–6].

Yet, in the meantime, another promising platform was introduced in the field, namely, the ICELL8 system (Takara Bio, USA), which uses a large-bore nozzle dispenser to distribute single cells of up to 150 μM in diameter, thereby suitable for entire adult cardiomyocytes [7]. By this, the well-based system (> 5,000 nanowells) enables size unbiased high-throughput single-cell analyses on adult murine and human hearts [8, 9]. Moreover, recent transcriptomic studies on fetal hearts have also employed scRNA-Seq as opposed to snRNA-Seq techniques. Thus, it can be safely assumed that scRNA-Seq will become and remain a widely applied method in cardiovascular research.

However, appropriate analyses of the respective data require a profound understanding of the underlying biological background and in this regard a proper adaption of the computational workflows. Regardless of the capture technique, captured cells are often stressed, damaged, or broken and some capture sites may contain multiple (doublets) or no cells at all. All these events refer to “low quality” cells, which may lead to misinterpretation of the data and thus need to be corrected. Common standard quality control parameters involve the number of genes, transcripts per cell, and the fraction of transcripts from mitochondrial genes (%mtRNA) [10]. In general, it is argued that an increased fraction of mtDNA-encoded genes hints at losses in cytoplasmic content, since in case of a broken cell membrane, cytoplasmic RNA will be lost, while RNA enclosed in the mitochondria will be retained. While cutoffs for transcripts and genes per cell are usually user-defined for each experiment or can be calculated individually by algorithms considering the standard deviation of transcript counts, a fixed threshold of 5% mitochondrial transcripts is commonly used as a standard in many scRNA-Seq studies.

Although this standard threshold is well known in the community, the origins of this arbitrary cut off are not easily traceable. In a supplementary note of a study on unique molecular identifiers for single-cell sequencing, Islam *et al.* plotted the total number of detected genes versus the fraction of cytoplasmic genes, which they defined as all non-mitochondrial and non-ribosomal RNA. Before, cells were stained with Red Fixable Dead Cell Stain for later validation of their bioinformatics quality controls. Based on the distribution of dead cells in this plot, they decided to set a threshold of max. 15% mitochondrial and ribosomal transcripts for their analysis. Although it was not distinguished between mitochondrial and ribosomal fractions, this publication is often cited in context to the usage of the mitochondrial fraction as a quality control parameter. Another study often mentioned in this context is the study of Ilicic *et al.* published three years later. They presented a generic approach for scRNA-Seq data processing and the detection of low quality cells, in which they identified functional categories that showed differences in expression levels between different types of low quality cells (multiple, damaged, empty) and high quality cells. In damaged cells, most Gene Ontology (GO) terms were downregulated, GO terms associated with mtDNA-encoded genes, however, were upregulated, supporting the view point of mitochondrial fraction as a marker for damaged cells. Although no exact threshold was recommended by the authors, this study is often referenced to justify the 5% mitochondrial transcripts threshold. Later, it was proposed in the “Guided Clustering Tutorial” of Seurat [11], one of the most common software packages for single-cell sequencing data analysis, and referred to by other bioinformatics software providers, such as Partek, thereby consolidating the application of this threshold.

However, this parameter is highly dependent on the tissue type. In the heart, mitochondrial transcripts account for almost 30% of total mRNA due to the high energy demand of cardiomyocytes [12]. Here, we demonstrate that applying the standard 5%-threshold results in an unacceptable exclusion of cardiomyocytes introducing a bias that particularly discriminates pacemaker cells. This effect is apparent for our in vitro generated induced-sinoatrial-bodies (iSABs), which are enriched for physiologically functional pacemaker cells, as well as in a public data set of ex vivo heart cells isolated from embryonal murine sinoatrial node tissue (Goodyer *et al.* [1]).

## Materials and methods

### Cell cultivation

The principle of differentiating pluripotent stem cells into “induced-sinoatrial-bodies” (iSABs) had been described by our group before [13]. After an initial cultivation in Dulbecco’s Modified Eagle’s medium–high glucose with 4 mM stabile l-glutamin (GIBCO), 10% FCS superior (Biochrom AG), 1% penicillin–streptomycin (GIBCO), 100 µM MEM non-essential amino acids (GIBCO), 1000 U/ml leukemia inhibitor factor (Phoenix Europe) diluted in aqua dest. with 0.1% BSA, and 100 µM β-mercapthoethanol (Sigma) for a minimum of 7 days, double transfected stem cells (TBX3 + MHCneoPGKhygro) were selected via addition of 10 µg/ml blasticidin (InvivoGen) and 250 µg/ml hygromycin (InvivoGen). Differentiation of selected cells started by generating embryoid bodies (EBs) in differentiation medium (IMDM medium (PAN-Biotech GmbH/Biochrom AG), 10% FCS superior (Biochrom AG), 1% penicillin–streptomycin, 100 µM MEM non-essential amino acids and 450 µM 1-thioglycerol and ascorbic acid) using a Spherical plate 5D (Kugelmeiers LTD). At day 3 of differentiation EBs were transferred onto 0.1% gelatine coated petri-dishes and adherently cultured at 37 °C and 5% CO_2_. Antibiotic selection of pacemaker cells with 400 µg/ml G418 (Biochrom) started on day 12 of differentiation. On day 20/21 of differentiation a transition to a floating cell culture was conducted by treating the cell layer with 6,000 U/ml collagenase IV (GIBCO) for 8 min at 37 °C, before centrifugation at 300×*g* for 5 min and resuspension in differentiation medium without ascorbic acid. Apoptotic cells were filtered out using a 40 µM cell strainer (Greiner Bio-One) and intact iSABs were floatingly cultured in ascorbic acid free differentiation medium until day 29.

### Single-cell RNA-sequencing

For single-cell analysis, iSABs were separated using the Primary Cardiomyocyte Isolation Kit (Thermo Fisher Scientific). Subsequently, cells were resuspended in PBS with 0.04% BSA and diluted to a concentration of 1,000 cells/μl. Cell viability amounted > 91% as assessed using the Cellometer Auto 2000 Cell Viability Counter. Single cells were then captured in droplet emulsions using the GemCode Single-Cell Instrument (10× Genomics) with a target output of 2,000 cells. Libraries for scRNA-Seq were constructed according to the 10× Genomics protocol using the GemCode Single-Cell 3′ Gel Bead and Library V3 Kit. Amplified cDNA and final libraries were evaluated on the 2100 Bioanalyzer instrument (Agilent) using a High Sensitivity NGS Analysis Kit (Advanced Analytical). Amplification yielded in 3.3 pg/µl cDNA and final libraries contained cDNA fragments with an average size around 450 bp. The subsequent sequencing was conducted on the HighSeq4000 Sequencing System using the HiSeq SBS and HiSeq PE Cluster Kit V4 (all Illumina, San Die-go, CA. USA). The isolation protocol for the embryonal sinoatrial node region from wild-type CD1 mouse hearts and the protocol for the subsequent scRNA-Seq can be viewed in the study of Goodyer *et al.* [1].

### Computational data analysis

Preprocessing of raw sequencing data from iSABs was conducted using tools of the Cell Ranger Software (v.6.1.0) as was the procedure in Goodyer *et al.* The Illumina sequencer’s base call files were demultiplexed into FASTQ files applying the implemented mkfastq pipeline. The scRNA-Seq fastq data files were then aligned with STAR (v.2.7) to the mm10 genome index (Ensembl release 98), annotated via GTF file, and grouped by barcodes and UMIs resulting in a feature-barcode matrix. Quality control involved the barcode ranking method and usage of the tool DropletUtils to exclude empty droplets and undetected genes. The subsequent data processing entailed standard normalization, identification of variable features, scaling, and dimensionality reduction by principal-component analysis (PCA) using the functions implemented in Seurat (v.3.2.2) similar for both data sets. Clusters were assigned to cardiac cell types based on classical marker genes. In contrast to the SAN data set, the complete omission of the threshold for mtDNA-encoded genes for the iSABs dataset led to clusters that could not be interpreted due to an excessively high proportion of mitochondrial transcripts (> 70%). Accordingly, the dataset was then subsetted by applying a low stringency threshold matching the mitochondrial fraction of the heart (30%) before assigning the generated clusters. Plots were generated using the DimPlot(), VlnPlot(), or FeatureScatter() function of Seurat, the latter implementing the calculation of Pearson correlation for the given features (here gene expression and %mtRNA). The entire code used for the computational data analysis is provided in our online available R script.

## Results

The iSABs dataset contained six clusters, all of which showed classical cardiomyocyte marker and four of which demonstrated a typical pacemaker signature (high expression of Hcn4, Hcn1, Tbx5, Shox2 etc.). Proportions of the clusters can be explored in the online supplementary material (Online Resource 1) and on our publicly available iRhythmics FairdomHub instance next to the experimental protocol and computational script.

Clusters of the SAN dataset were assigned to cardiac cell types based on classical marker genes and adopting Goodyers’ annotation. Although we implemented the more widely preferred uniform manifold approximation and projection (UMAP) method for dimensionality reduction and visualization instead of a t-distributed stochastic neighbor embedding (t-SNE) based visualization as used by Goodyer *et al.,* we obtained a comparable plot for the dataset (Online Resource 2). Minor differences in the clustering results compared to the original study may be attributed to slightly different preprocessing approaches. Whereas Goodyer *et al.* also omitted the threshold for mtDNA-encoded genes in their study, they additionally scaled their data on the percentage of ribosome genes, and Rn45s expression. Subsequent differences involve the total number of cells (6,322 compared to 5,919 in the original study) and the proportion of cells in the individual clusters (e.g., 181 cells in the SAN cluster compared to 200 cells in the original study) but are not critical for downstream analyses.

**Fig. 1:**
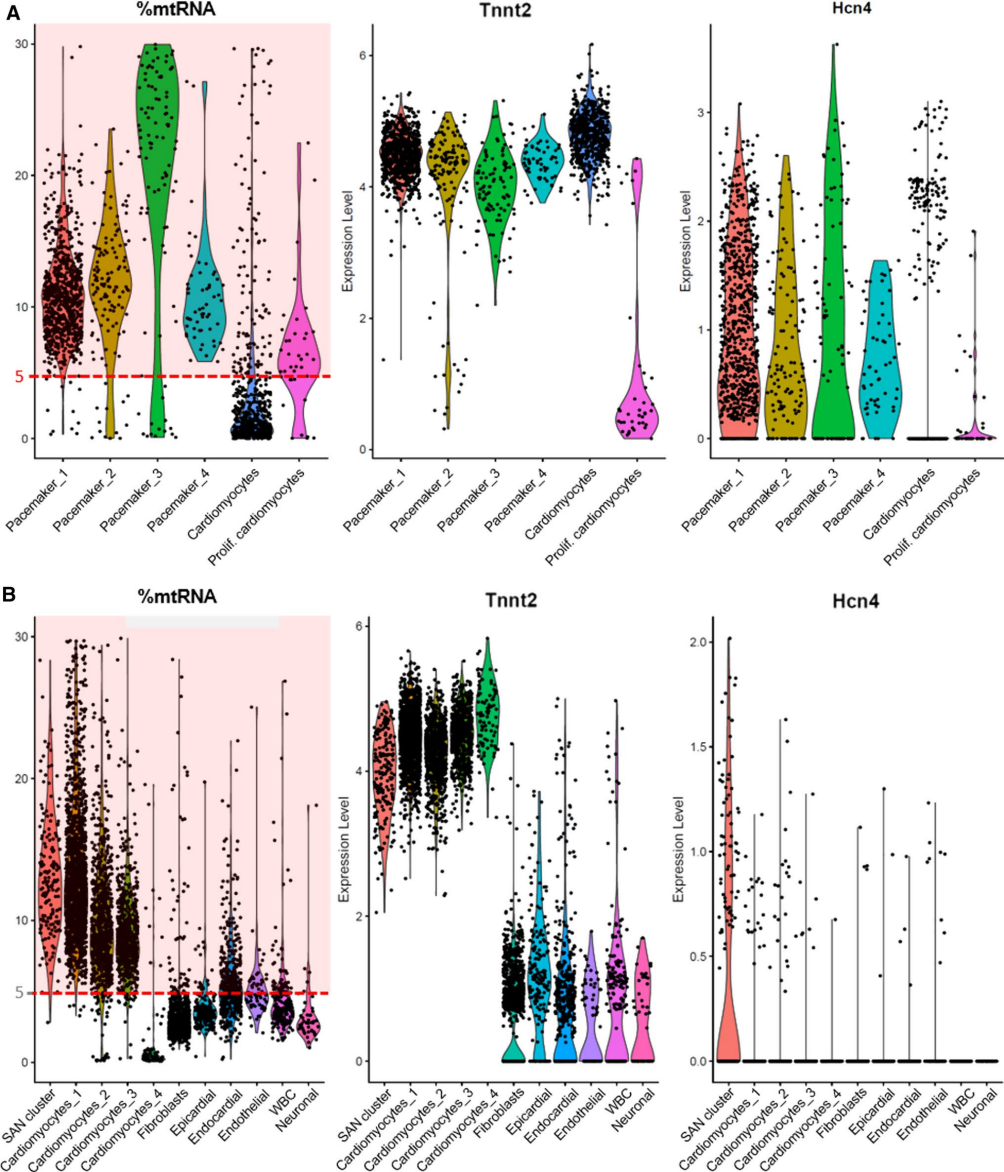
Violin plot for mitochondrial gene transcripts (%mtRNA), cardiac marker (Tnnt2), and pacemaker marker (Hcn4) for identified cell clusters. **A** In iSABs, most pacemaker cells exceed 5% mtRNA; **B** almost all cells of the SAN cluster of murine sinoatrial node tissue exceed 5% mtRNA. Results obtained from a data reanalysis of Goodyer *et al.* [1]

Plotting the fraction of mitochondrial gene transcripts, we found that all pacemaker subpopulations in the iSABs dataset were characterized by %mtRNA values above the 5%-threshold (Fig. 1A). Applying this quality control parameter for filtering would result in a loss of these cells for downstream analysis, thereby generating a biased image of the biological sample. Likewise, the SAN cluster of the Goodyer dataset was characterized by similar %mtRNA values to our in vitro pacemaker cells likely exposing the same bias (Fig. 1B).

While fibroblasts, epicardial cells, and neuronal cells are not much affected by the mitochondrial fraction quality control parameter, a fair amount of endothelial and endocardial cells would not pass the standard threshold and most cardiomyocyte clusters contain a majority of cells with mtRNA > 5%. This discrimination is most severe for pacemaker cells of the SAN cluster, where only 3 out of 181 cells would pass the 5%-threshold.

**Fig. 2:**
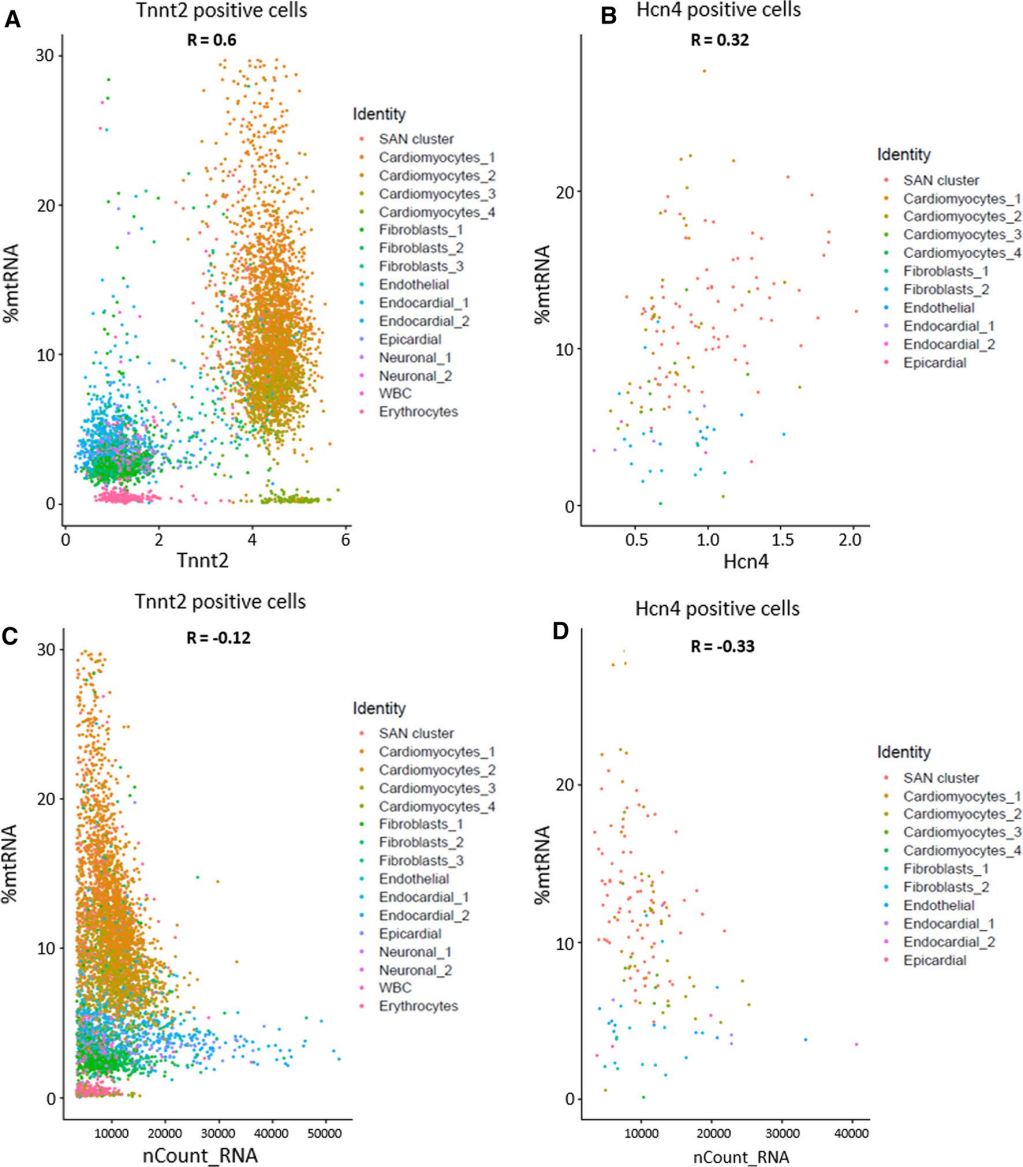
Scatter plots illustrating the correlation of %mtRNA and other parameters. **A** In Tnnt2 positive cells, there is a positive correlation between %mtRNA and the cardiac marker Tnnt2. **B** In Hcn4 positive cells, there is a positive correlation between %mtRNA and the pacemaker cell marker Hcn4. **C** In Tnnt2 positive cells, there is a weak negative correlation between the fraction of mitochondrial gene transcripts and the total number of gene transcripts per cell. **D** In Hcn4 positive cells, there is a negative correlation between the fraction of mitochondrial gene transcripts and the total number of gene transcripts per cell

To precisely examine this bias, we characterized the correlation of %mtRNA along with our marker genes Tnnt2 and Hcn4. In this regard, we subsetted the SAN dataset to exclude all cells without gene expression information for Tnnt2 or Hcn4, respectively, before calculating the Pearson correlation. As expected, we found a positive correlation between %mtRNA and the cardiomyocyte marker Tnnt2 (*R* = 0.6, Fig. 2A), which is plausible, because cardiomyocytes have a high energy demand and, therefore, bear a large number of mitochondria. Notably, a positive correlation could also be shown for Hcn4 and %mtRNA, indicating that Hcn4 expression is associated with higher mitochondrial fractions (*R* = 0.32, Fig. 2B).

To exclude that this correlation is simply the result of general higher transcript numbers in cells with high %mtRNA, we checked the relation of these parameters. There was a very low correlation for the Tnnt2 positive cells (*R* = − 0.12, Fig. 2C) and the data for Hcn4 positive cells implied that rather the opposite applies and a high fraction of mitochondrial transcripts was associated with lower total transcript counts (*R* = − 0.33, Fig. 2D). The fact that despite this trend to lower total transcript counts, cells with high mitochondrial fraction show higher counts for Hcn4 transcripts suggests that the %mtRNA can serve as a further surrogate marker for pacemaker cells.

## Discussion

A crucial step in any scRNA-Seq analysis is the cell quality control. This step is supposed to exclude low quality cells and doublets that might impair the downstream analyses and is typically based on three covariates: the total number of transcripts per cell (count depth), the total number of detected genes per cell, and the fraction of transcripts from mitochondrial genes. In addition, using a PCA is a basic and broadly applicable approach to identify outlier cells but requires general bioinformatics knowledge to be applied properly. Moreover, many groups rely on the implementation of ERCC RNA spike-ins and compare the ratio of reads mapped to spike-ins against the number of total mapped reads to detect endogenous transcript loss. However, 10× Genomics for instance does not recommend this approach for their assays [14], leaving the experimentalist with the initially mentioned standard parameters.

Applying respective filters demands special caution, since there can be biological interpretations for aberrant values. For example, low transcript or gene numbers may be characteristic of quiescent cell populations and high counts may arise from large cells. Accordingly, thresholds are usually user-defined for each experiment individually based on specific guidelines [10, 15]. For low-count filtering, the transcripts per cell are visualized and a threshold is applied, where count depths start to decrease rapidly. For high‐count filtering, it is recommended that the proportion of filtered cells should not exceed the expected doublet rate. To filter out genes that are expressed in only a few cells and, therefore, rendered irrelevant, the threshold is adjusted to the minimum cell cluster size of interest plus some leeway for dropout effects. Notably, there are no such recommendations for the adjustment of the threshold for mtDNA-encoded genes, despite an awareness of biological interpretations for a high fraction of mitochondrial transcripts, such as involvement in respiratory processes.

A fixed threshold of 5% mitochondrial transcripts has established as standard and is set as default in several software packages for scRNA-Seq analysis [11]. Moreover, aware that the whole heart in general and cardiomyocytes in particular show an average fraction of mitochondrial transcripts significantly higher than 5%, Osorio *et al.* still concluded in their systematic meta-analysis that 5% mtRNA is an appropriate threshold for murine tissues and that omitting this filter may lead to erroneous biological interpretations of scRNA-Seq data [16].

Contrarily, we found that for murine cardiac tissue sticking to the 5% threshold causes biased results as distinct cell types are affected by this filter to varying degrees. For example, a large proportion of cardiomyocytes of the SAN region was shown to have fractions of mitochondrial transcripts above the threshold, while only very few fibroblasts exceed this limit. Moreover, we demonstrated here that a high fraction of transcripts from mitochondrial genes also represents a marker for pacemaker cells and that an employment of the 5% mtRNA filter results in the elimination of this population from the dataset.

Among other cardiac cell populations, a small number of white blood cells demonstrated a relatively high fraction of mitochondrial transcripts. Notably, mitochondrial biogenesis was shown to be functionally connected with the immune response. In particular, rapid changes including an increase in number and mtDNA content of mitochondria have been observed upon T cell activation [17]. Such effects might be the underlying reason for some white blood cells to exceed the 5% limit and support the idea that increased metabolic activity results in higher fractions of mitochondrial transcripts. Inflammatory events for example in context with myocardial infarcts are of high interest in the cardiovascular field. In summary, the results of this study point at limitations of the standard threshold for mtDNA-encoded genes for investigations on the heart.

More specifically, we herewith have demonstrated for the first time that scRNA-Seq data from pacemaker cells, that are naturally rare, are particularly affected by a lack of proper adaption of quality control measures. This implicates that at least for cardiovascular research it will be essential to empirically determine the best-suited value for the mtRNA-threshold for each analysis individually to avoid the introduction of biases. Going even further, we do recommend to completely omit the fraction of mitochondrial transcripts as a default quality control parameter whenever possible. However, the feasibility of omitting this filter completely depends highly on the overall quality of the biological samples and needs to be evaluated for each individual experiment.

The raw data set of Goodyer *et al.*, which we have re-analyzed, proves that it is possible to omit the mtRNA-threshold completely without negative impacts on the analysis outcome. Yet, a high fraction of mitochondrial reads can complicate the cluster annotation and hamper some downstream analyses. To avoid these negative effects it is possible to preclude mitochondrial genes from the count matrix for later steps. In a recent study on adult human heart, Wang *et al.* retained all cells with mitochondrial transcripts < 72% but subsequently removed the respective mitochondrial genes from the count matrix [9]. Unfortunately, the authors provide only this manipulated count matrix instead of the raw data. Thus, it was not possible to verify our findings on the mitochondrial fractions in pacemaker cells with this human data set. In this context, we advocate that it becomes common practice to provide actual raw data formats thereby enabling to customize the quality control for each analysis.

In general, a distinction between signal and noise of cells within dedicated clusters can be facilitated through current normalization techniques (e.g., SCT) [18, 19]. Alternatively, specialized tools, such as EMBEDR, recover the ability to separate signal and noise in dimensionality reduction outputs, such as tSNE and UMAP representations, which is essential for the subsequent utilization in quantitative analyses [20]. The obtained embedding quality is made available as a cellwise, interpretable *p* value that has meaning across datasets. Besides these classical bioinformatics approaches, an alternative means to assess the quality of cardiomyocytes and other cells is to visually inspect their morphological features. For example, using the Icell8 platform allows for basic microscopic examination of the cells and detection of stainings in three channels (e.g., for dead-life-assays). As most low quality cells are visibly damaged, cell imaging helps to identify a large proportion of low quality cells. However, it is not feasible for all single-cell sequencing methods and relatively inefficient and time-consuming for larger cell counts.

To avoid the use of arbitrary %mtRNA thresholds, Ma *et al.* suggested an unsupervised method for optimization of quality control parameters, called EnsembleKQC [21]. The threshold is based on a function of the distribution of the data and represents a more objective method for the quality control of biological samples. However, this approach comes with two limitations. On the one hand, a corrupted sample with a large proportion of damaged cells will produce a data set in which most cells demonstrate increased mitochondrial fractions. Based on these increased values, the optimized threshold might be inappropriately high. On the other hand, tissue samples that are more heterogeneous might demonstrate an unequal distribution of the data. Cell types with unusually high or low mitochondrial fractions might be excluded for their “abnormal” characteristics.

Very recently, another data-driven approach was proposed in a preprint of Hippen *et al. *[22]. Applying mixture models in a probabilistic framework their QC metric (miQC) combines both the fraction of mitochondrial transcripts and the number of detected genes to computationally predict low quality cells. Using a tumor sample, they demonstrate that miQC preserves more cells within identified clusters and minimizes sub-population bias, compared to a uniform threshold approach that can result in a disproportionate exclusion of certain cell populations as demonstrated in this manuscript. By now, miQC might currently be the most appropriate tool to control the quality of scRNA-seq of heart tissue and other heterogeneous tissues in a more objective manner. In general, it is recommended to consider several parameters in conjunction to gain a more detailed overview on the overall quality of the data.

## Conclusion

As one method of quality control the mitochondrial fraction is a typical threshold parameter to exclude low quality cells. However, for cardiac tissue, the standard 5% filter eliminates a large proportion of cardiomyocytes and almost all pacemaker cell data. Thus, we recommend omitting this parameter for scRNA-seq in cardiovascular applications whenever possible. As an increasing number of tools for processing of scRNA-seq data are developed, we believe it will soon be feasible to handle the %mtRNA as a cellular feature instead of employing it mainly for quality control purposes.

## Supplementary Information

Below is the link to the electronic supplementary material.

Supplementary file1 (PNG 144 kb)

Supplementary file2 (PNG 432 kb)

## Data Availability

Additional UMAP plots for both datasets (Online Resource 1: UMAP projection of iSABS clusters, Online Resource 2: UMAP projection of SAN clusters from Goodyer *et al.*) are provided on our publicly available iRhythmics FairdomHub instance. Raw Fastq files from scRNA-Seq of iSABs are available in the Gene Expression Omnibus (GEO) repository (Accesion number: GSE174233).

## Code availability

The computational R script is available online at iRhythmics FairdomHub (https://fairdomhub.org/studies/841).
