# Supplementary figures and images for: Quality control in scRNA-Seq can discriminate pacemaker cells: the mtRNA bias
Source: Cell Mol Life Sci. 2021 Aug 24;78(19-20):6585–92. doi: 10.1007/s00018-021-03916-5 (PMC8558157; doi:10.1007/s00018-021-03916-5)

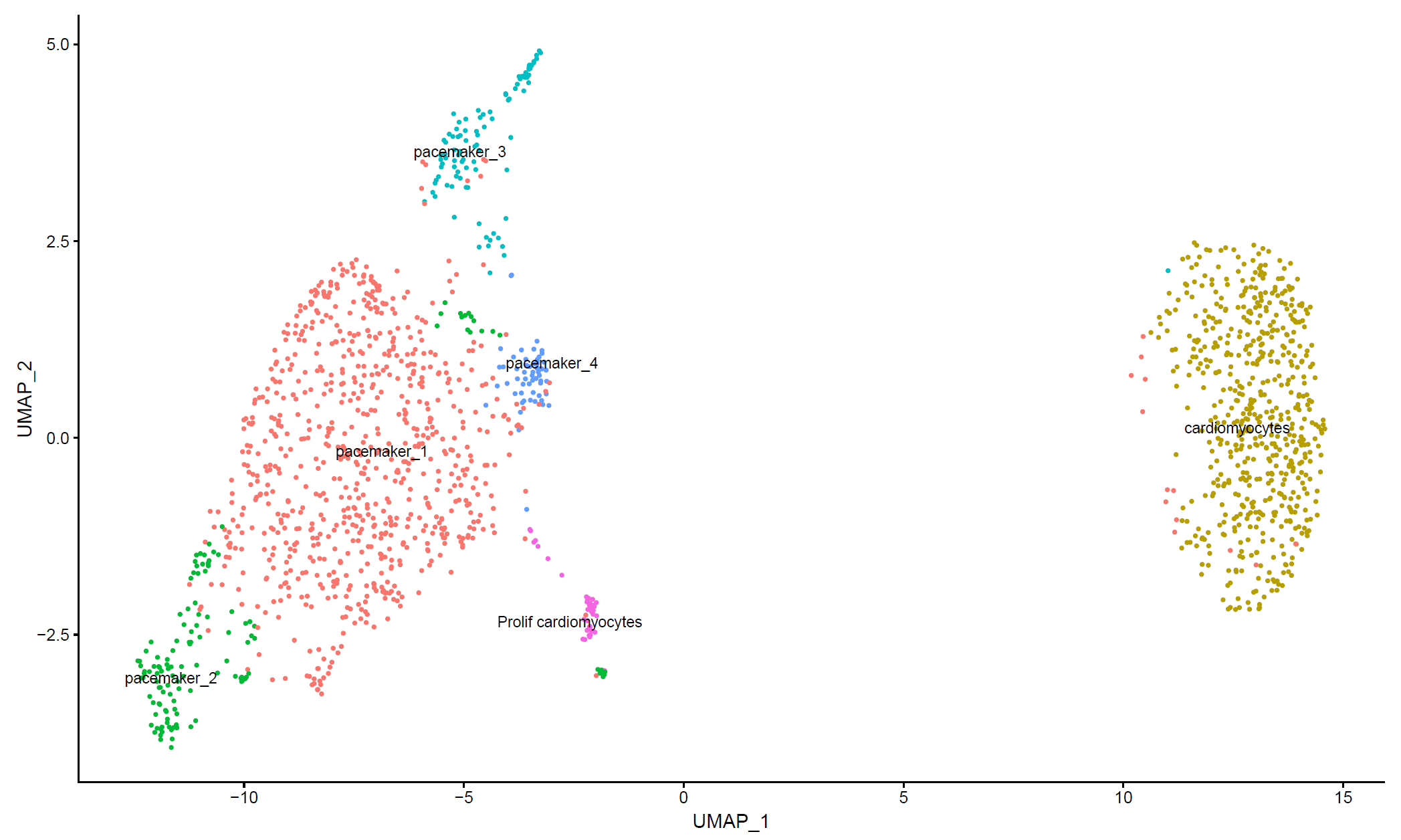

Supplement: Supplementary file 1 — Supplementary file1 (PNG 144 kb) [file 18_2021_3916_MOESM1_ESM.png]

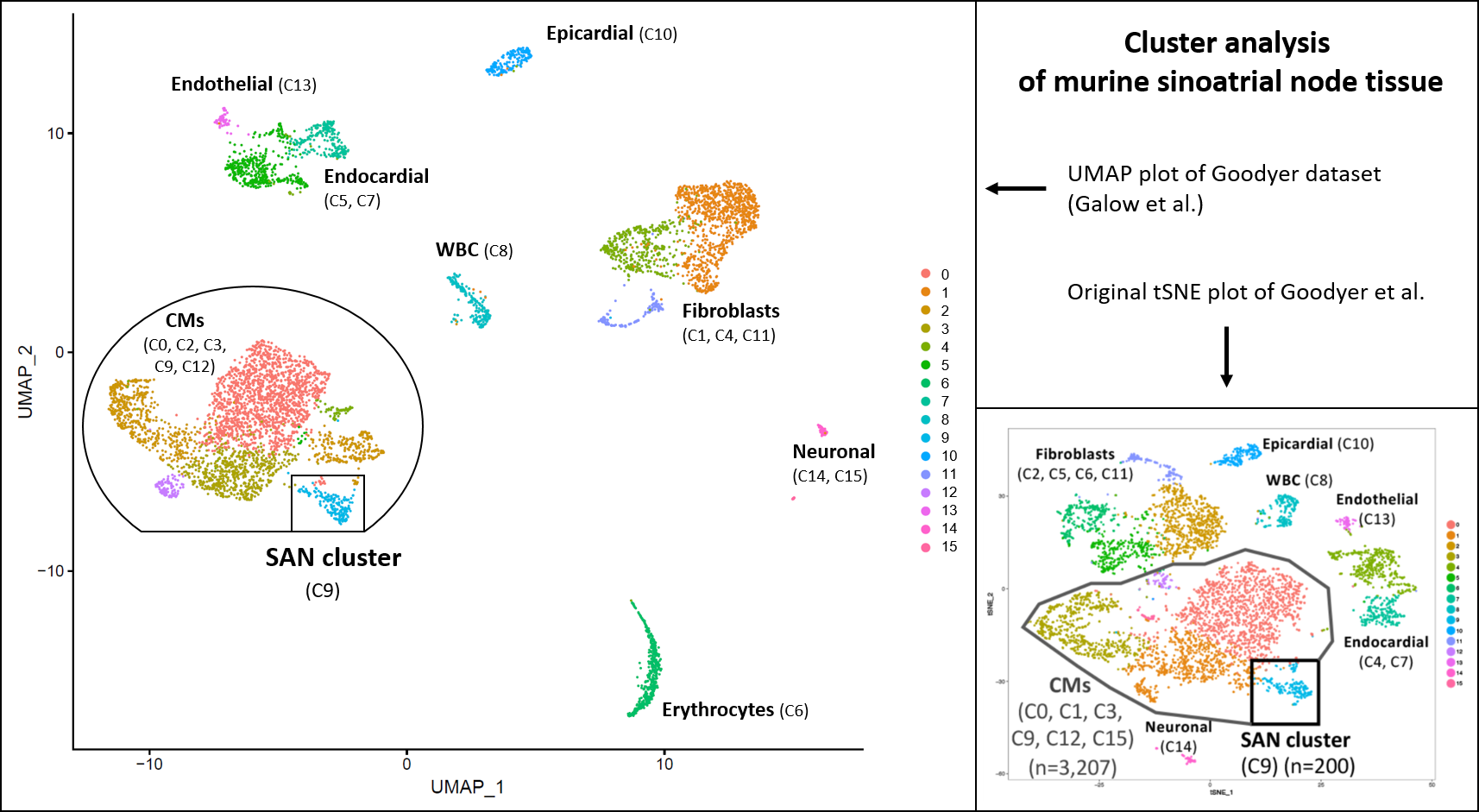

Supplement: Supplementary file 2 — Supplementary file2 (PNG 432 kb) [file 18_2021_3916_MOESM2_ESM.png]
